# Microbial Community Response of an Organohalide Respiring Enrichment Culture to Permanganate Oxidation

**DOI:** 10.1371/journal.pone.0134615

**Published:** 2015-08-05

**Authors:** Nora B. Sutton, Siavash Atashgahi, Edoardo Saccenti, Tim Grotenhuis, Hauke Smidt, Huub H. M. Rijnaarts

**Affiliations:** 1 Environmental Technology, Wageningen University, Wageningen, The Netherlands; 2 Laboratory of Microbiology, Wageningen University, Wageningen, The Netherlands; 3 Laboratory of Systems and Synthetic Biology, Wageningen University, Wageningen, The Netherlands; Sun Yat-Sen University, CHINA

## Abstract

While *in situ* chemical oxidation is often used to remediate tetrachloroethene (PCE) contaminated locations, very little is known about its influence on microbial composition and organohalide respiration (OHR) activity. Here, we investigate the impact of oxidation with permanganate on OHR rates, the abundance of organohalide respiring bacteria (OHRB) and reductive dehalogenase (*rdh*) genes using quantitative PCR, and microbial community composition through sequencing of 16S rRNA genes. A PCE degrading enrichment was repeatedly treated with low (25 μmol), medium (50 μmol), or high (100 μmol) permanganate doses, or no oxidant treatment (biotic control). Low and medium treatments led to higher OHR rates and enrichment of several OHRB and *rdh* genes, as compared to the biotic control. Improved degradation rates can be attributed to enrichment of (1) OHRB able to also utilize Mn oxides as a terminal electron acceptor and (2) non-dechlorinating community members of the *Clostridiales* and *Deltaproteobacteria* possibly supporting OHRB by providing essential co-factors. In contrast, high permanganate treatment disrupted dechlorination beyond *cis*-dichloroethene and caused at least a 2–4 orders of magnitude reduction in the abundance of all measured OHRB and *rdh* genes, as compared to the biotic control. High permanganate treatments resulted in a notably divergent microbial community, with increased abundances of organisms affiliated with *Campylobacterales* and *Oceanospirillales* capable of dissimilatory Mn reduction, and decreased abundance of presumed supporters of OHRB. Although OTUs classified within the OHR-supportive order *Clostridiales* and OHRB increased in abundance over the course of 213 days following the final 100 μmol permanganate treatment, only limited regeneration of PCE dechlorination was observed in one of three microcosms, suggesting strong chemical oxidation treatments can irreversibly disrupt OHR. Overall, this detailed investigation into dose-dependent changes of microbial composition and activity due to permanganate treatment provides insight into the mechanisms of OHR stimulation or disruption upon chemical oxidation.

## Introduction

Anthropogenic contamination of soil and groundwater with organic chlorinated solvents such as tetrachloroethene (PCE) and trichloroethene (TCE) requires innovative, efficient, and cost-effective remediation technologies. To this end, increasing emphasis is being placed on *in situ* technologies which are less invasive and costly than more traditional *ex situ* treatments. Approaches including *in situ* chemical oxidation (ISCO) and *in situ* bioremediation (ISB) can effectively and efficiently remediate locations without significant disruption of above ground activities [1, 2].

Chemical treatment of chlorinated solvents *in situ* is often performed to rapidly oxidize high concentrations of contaminants [2]. Chemical oxidants such as permanganate, persulfate, hydrogen peroxide, and ozone can efficiently decrease contaminant mass more quickly than, for example, biological treatment. However, once the oxidant has finished reacting, rebound of aqueous contaminant concentrations may occur, either due to dissolution of residual pure product or desorption of solvents sorbed to the soil matrix [3–5]. In such instances, a biopolishing step following ISCO could be advantageous to remove residual contaminants and ensure complete remediation.

Microbial capacity to biodegrade PCE has been exploited in the development of ISB technologies for treatment of chlorinated solvent contaminated locations. Under anaerobic conditions, PCE is degraded by sequential substitution of chlorine atoms for hydrogen atoms to produce, upon complete dechlorination, ethene in an energy conserving process known as organohalide respiration (OHR; [6]). Full scale ISB treatment of PCE contaminated locations often includes subsurface amendment with electron donor and nutrients known as biostimulation; in some cases bioaugmentation with organohalide respiring bacteria (OHRB) able to perform OHR of the target compound is required to ensure complete dechlorination [7].

A restricted but growing number of OHRB have been enriched and isolated in pure culture with the capacity to reductively dechlorinate PCE to TCE, *cis*-dichloroethene (DCE), vinyl chloride (VC) and finally ethene, with *Dehalococcoides mccartyi* being the only organism known to perform full transformation of PCE to ethene [8]. A variety of biomolecular techniques has been developed to specifically monitor OHRB and their reductive dehalogenase (*rdh*) genes. These tools can be used to quantitatively verify biodegradation capacity by monitoring changes in target abundance, thereby assisting in contaminated site management, for example, by indicating whether or not bioaugmentation may be necessary [9, 10].

While axenic cultures of OHRB provide valuable fundamental knowledge about the biochemistry and physiology of these bacteria, OHRB exhibit higher dechlorination rates when present as members of microbial communities. This is in part due to the fastidious nature of key OHRB. In particular, known strains of *D*. *mccartyi* have been shown to have notable nutritional requirements. Recent metagenomic analysis of enrichment cultures revealed the important role of non-dechlorinators in fulfilling such growth requirements of OHRB [11, 12]. These community members produce fermentation intermediates such acetate and hydrogen, provide essential co-factors (including corrinoids and methionine) and perform oxygen scavenging, while profiting from the removal of toxic chlorinated compounds by OHRB [11, 13, 14]. Building upon this knowledge, a number of studies have yielded insights on microbial community composition and syntrophic interactions, supporting chlorinated solvent degradation in the lab [15–17] and in the field [18, 19].

Whereas ISB has been successfully implemented following ISCO for the remediation of aliphatic and aromatic hydrocarbons [20–23], coupling chemical oxidation with an anaerobic bioremediation step for the treatment of chlorinated solvents is more challenging. In addition to the direct oxidative stress inflicted by the oxidant, increased oxidation-reduction potential associated with chemical oxidation may be detrimental to strictly anaerobic OHRB or other non-dechlorinating community members. A number of studies have examined the regeneration of OHR capacity following chemical oxidation at batch [24], column [25, 26], and field scales [9, 27]. These investigations revealed threshold oxidant concentrations [24] and the necessity for amendments such as electron donor and bioaugmentation to support biodegradation [25, 26]. In some instances, the abundances of OHRB and *rdh* genes were measured to quantify the impact of chemical oxidation on and subsequent regeneration of OHR capacity [9, 24, 26]. However, these investigations do not provide information on the non-dechlorinating community members that were shown to play indispensable roles in supporting robust and stabile dechlorinating consortia [11, 12]. In addition to the interactions described above, the entire microbial community plays a role in attenuation of oxidation-reduction potential following chemical oxidation to ensure regeneration of OHR activity.

Here we investigated the impact of chemical oxidation with permanganate on a PCE degrading consortium using both quantitative PCR (qPCR) and sequencing of 16S rRNA gene fragments. By determining changes in OHRB and *rdh* gene abundance as well as microbial community structure following chemical oxidation, this study improves our insight into the impact of ISCO on microbial communities and their subsequent regeneration following oxidative stress. To our knowledge, the results presented here are a new step towards better understanding and thus application of biopolishing following chemical oxidation.

## Materials and Methods

### 2.1 Microcosm Setup

A total of fourty microcosms were prepared in 125 mL glass serum bottles with Viton stoppers (Rubber b.v., The Netherlands) using a PCE dechlorinating enriched culture, maintained on lactate and PCE at 25°C prior to this study. Prior to inoculation, a final concentration of 10 mM sodium lactate and 2.0 μmol of PCE, both dissolved in anoxic water, were added to the medium [28]. Bottles were inoculated with 5 mL of the enriched culture, thereby reaching a final volume of 50 mL, corresponding to a PCE concentration of 40 μmol/L. Microcosms were incubated at 25°C shaking at 120 rpm prior to treatment with chemical oxidants; chlorinated ethenes were measured in 5 microcosms to ensure OHR activity. Two microcosms were sacrificed for DNA sampling at the start of the experiment (labeled “S”) to determine the initial microbial community, as described below.

### 2.2 Treatment and sampling

Four different conditions were tested with nine replicate microcosms prepared for each treatment: a biotic control which did not receive permanganate and three chemical oxidation treatments with low (25 μmol), medium (50 μmol) and high (100 μmol) permanganate doses. The biotic, low and medium microcosms received PCE additions of 0.86 μmol on days 0, 3, 4, 7, 9, 10, 13, 16, and 17 and permanganate was applied on days 0, 4, 7, 10, 13, and 17 (see Table 1 for details on timing of PCE and permanganate additions and sampling). Sampling for DNA extraction from these microcosms was performed at days 7, 13 and 21 that were considered as the end of treatment cycles 1, 2, and 3, respectively. To ensure sufficient biomass for molecular analyses, each time two microcosms were randomly selected and sacrificed for DNA extraction. Microcosms receiving high permanganate doses showed a larger disruption of OHR activity after the first permanganate addition (Table 1). Hence, permanganate treatments were only performed on days 0, 13, and 33 and additional incubation time was considered to allow regeneration of the microbial community (Table 1). Sampling for the first molecular analyses was performed on day 13. A second DNA sampling was performed on day 21 to temporally coincide with sampling performed in biotic, low and medium treatment microcosms. The third DNA sampling was performed on day 33 followed by the third and final permanganate treatment. Hereafter, these microcosms were monitored over an extended period to track possible regeneration of OHR activity. Only one microcosm (H7) resumed OHR activity, and was sampled on day 163, whereas the two remaining microcosms were sacrificed for DNA isolation on day 246.

**Table 1:** Treatment and sampling scheme of microcosms. Biotic control (“B”), low (“L”; 25 μmol) or medium (“M”; 50 μmol) permanganate treatments (above) and high (“H”; 100 μmol) permanganate treatment (below) are given. DNA samples were taken in most cases in duplicate, with microcosm numbers (in bold) corresponding to Table 2. PCE spiking (0.86 μmol) and permanganate (PM) dosing are indicated (x).

	Days	0	1	2	3	4	5	6	7	8	9	10	11	12	13	14	15	16	17	18	19	20	21	22	. . .	33	. . .	163	. . .	246
Biotic, Low, Medium	DNA	**S1+2**	** **	** **	** **	** **	** **	** **	**B, L, M1+2**	** **	** **	** **	** **	** **	**B, L, M 3+4**	** **	** **	** **	** **	** **	** **	** **	**B, L, M5+6**							
	PM (25 or 50 μmol)	x				x			x			x			x				x											
	PCE (0.86 μmol)	x			x	x			x		x	x			x			x	x											
High	DNA	**S1+2**													** H1+2**								**H3+4**	** **		** H5+6**		**H7**		**H8+9**
	PM (100 μmol)	x													x											x				
	PCE (0.86 μmol)	x			x										x											x				

Lactate measurements in all microcosms receiving permanganate were used to assess when amendment was required to maintain lactate concentrations around 10 mM. Headspace analysis of PCE, TCE, *cis*-DCE, VC, and ethene concentrations was always performed one day after addition of PCE and regularly during the first 33 days of the experiment; thereafter, measurements were less frequent.

### 2.3 Chemical Analyses

Lactate was measured in liquid samples on an HPLC with an organic acids column (Ion 300) and refractive index detector. Samples for Mn^2+^ measurements were collected in 2 mL microcentrifuge tubes, acidified with concentrated HNO_3_, and determined by inductively coupled plasma-optical emission spectroscopy. PCE, TCE, *cis*-DCE, VC and ethene were measured in the headspace. PCE, TCE, and *cis*-DCE were extracted for 2 minutes with a 100 μm polydimethylsiloxane (PDMS) coated fiber. The compounds were injected at 250˚C on a Fisions 8000 series GC with a CP-Sil8 column (25 m x 0.53 mm x 5.0 μm) using helium as the carrier gas and detected using a Flame Ionization Detector (FID). The program started at 50˚C, ramped at 20˚C/min to 140˚C, and remained at 140˚C for the final 1.5 min. For VC and ethene, 100 μL of headspace was sampled with a glass syringe and directly injected on a HP6890 series GC with a CP PoraBond Q column (25 m x 0.53 mm x 10 μm). The temperature program was isothermal at 60˚C and detection was with a FID at 300˚C. Rates of PCE and TCE conversion were calculated by comparing the total mass of either PCE or TCE measured one day after each PCE spike to the sum of those measured and spiked the previous day (S1 Fig).

### 2.4 DNA sampling and extraction

Samples for molecular analyses of total DNA were collected by sacrificing two replicate microcosms of each condition. The total volume of each microcosm was vacuum filtered over a separate sterile 0.22 μm membrane filter (Milipore, USA). Each filter was folded and placed in a sterile microcentrifuge tube, snap-frozen in liquid nitrogen, and stored at -20°C until DNA extraction. The volume of filtered liquid was measured using a graduated cylinder to allow calculation of gene copy numbers/mL. DNA extraction was performed using the FastDNA Spin Kit for Soil (MP Biomedicals, USA) according to manufacturer’s instructions; the filter was sliced into pieces prior to placement in the bead beater to aid extraction. DNA was eluted with DNase/Pyrogen-Free water, controlled for quantity and quality using a Nanodrop spectrophotometer (Thermo Scientific, Germany), and stored at -20°C.

### 2.5 Quantitative PCR

Quantitative PCR (qPCR) was performed to measure total bacteria, total archaea, and specific OHRB based on the 16S rRNA gene and *rdh* genes as described previously [9, 29]. Assayed OHRB were *D*. *mccartyi*, *Geobacter*, *Desulfitobacterium*, *Dehalobacter*, *Sulfurospirillum*, and assayed *rdh* genes were *tceA*, *bvcA*, and *vcrA*, encoding TCE and VC reductive dehalogenases in *D*. *mccartyi*, respectively. Ten-fold dilutions of total DNA extracts were assayed in triplicate using an iQ5 SYBR Green Supermix kit on the iQ5 iCycler (Biorad, The Netherlands). Assayed targets and primer sequences have been described previously [9, 29] and are summarized in S1 Table. Gene copy numbers are expressed as copy/mL culture. To account for natural shifts in abundance due to incubation, results were normalized to the biotic control by dividing the observed abundances in permanganate-treated microcosms at a given time point to that observed in the biotic control.

### 2.6 Bacterial 16S rRNA gene amplicon pyrosequencing

Bacterial 16S rRNA gene fragments were amplified using barcoded primers covering the V1-V2 region of the bacterial 16S rRNA gene. The forward primer consisted of the 27F-DegS primer (5´- GTTYGATYMTGGCTCAG- 3´) [30] appended with the titanium sequencing adaptor A (5´- CCATCTCATCCCTGCGTGTCTCCGACTCAG- 3´) and an 8 nucleotide sample specific barcode [31] at the 5´ end. An equimolar mix of two reverse primers was used i.e. 338RI (5´- GCWGCCTCCCGTAGGAGT- 3´) and 338RII (5´- GCWGCCACCCGTAGGTGT- 3´) [32] that carried the titanium adaptor B (5´- CCTATCCCCTGTGTGCCTTGGCAGTCTCAG- 3´) at the 5´ end. Sequences of both titanium adaptors were kindly provided by GATC Biotech (Konstanz, Germany). The PCR mix (100 μl final volume) contained 20 μl of 5× HF buffer (Finnzymes, Vantaa, Finland), 2μl PCR Grade Nucleotide Mix (Roche Diagnostic GmbH, Mannheim, Germany), 1μl of Phusion hot start II High-Fidelity DNA polymerase (2U/μl; Finnzymes), 500 nM of the reverse primer mix and the forward primer (Biolegio BV, Nijmegen, The Netherlands), 2 μl (40 ng) template, and 65 μl nuclease free water. PCR was performed using the following conditions: 98°C for 30 s to activate the polymerase, followed by 30 cycles of denaturation at 98°C for 10 s, annealing at 56°C for 20 s, elongation at 72°C for 20 s, and a final extension at 72°C for 10 min. Five μl of the PCR reactions, were analyzed by 1% (w/v) agarose gel electrophoresis, containing 1× SYBR Safe (Invitrogen, Carlsbad, CA, USA) to verify the right length of the amplicons (approximately 450 bp). PCR products were purified using GeneJET PCR purification kit (ThermoScientific, Germany) according to the manufacturer’s instructions. The DNA concentration of the purified amplicons was measured using NanoDrop, and amplicons were mixed in equimolar amounts and run again on an agarose gel prior to excision and purification by using a DNA gel extraction kit (Millipore, Billerica, MA, USA). Amplicons obtained from the 24 samples were analysed simultaneously with 30 unrelated samples by pyrosequencing on half a plate using an FLX genome sequencer in combination with titanium chemistry (GATC-Biotech, Konstanz, Germany).

### 2.7 Analysis and interpretation of the pyrosequencing data

Pyrosequencing data was analysed using the QIIME 1.8.0 pipeline [33]. Sequence reads were initially filtered using default parameters and denoised [34] for removing low quality or ambiguous reads. Chimeric sequences were then removed from pre-processed data from the dataset using UCHIME [35]. From the remaining set of high quality 16S rRNA gene sequences, operational taxonomic units (OTUs) were defined at a 97% identity level. A representative sequence from each OTU was aligned using PyNAST [36]. The taxonomic affiliation of each OTU was determined at an identity threshold of 97% using uclust [35] and SILVA 111 database as a reference [37]. The complete dataset is available at the European Bioinformatics Institute (www.ebi.ac.uk) under accession number PRJEB8632.

### 2.8 Statistical analysis

In order to interpret the changes in microbial communities to experimental variables, redundancy analysis (RDA) was used as implemented in the CANOCO 5 software package (Biometris, Wageningen, The Netherlands). The experimental variables tested were number of permanganate additions labeled as doses, the cumulative permanganate addition (as μmol), length of incubation as days, and cumulative PCE addition as number of spikes. A Monte Carlo permutation test based on 499 random permutations was used to determine which of the experimental variables significantly contributed to the observed variance in the composition of microbial communities at the order level. Orders of at least 0.05 relative abundance in any sample were included in the analysis (S2 Table). The community structure was visualized via ordination triplots with scaling focused on intersample differences.

Additionally, a linear mixed model was fitted to order level relative abundances above the 0.05 level. The 29 bottles were considered to be randomly assigned to the different levels of the fixed experimental factor described above. Additionally, the type of treatment (biotic control, low, medium or high permanganate dosage) was also included as fixed factors. The bottles were considered as random term and interactions were not estimated. Calculations were performed using the REML procedure of Genstat 16^th^ SP1 (VSN International, Hemel Hempstead, UK).

## Results

### 3.1 Regeneration of OHR activity following permanganate treatment

The impact of chemical oxidation on OHR activity was investigated by comparing microcosms receiving permanganate treatment with the biotic control without chemical treatment. Permanganate doses are relatively low compared to field application [27], but were similar to those applied in column [26] and batch [24] experiments. The low oxidant concentrations resulted in very little PCE oxidation, as the majority of permanganate was consumed by other reduced constituents. Low chemical oxidation efficiency of PCE was deemed irrelevant, as the goal of this research was to determine the impact of permanganate treatment on the microbial community.

**Fig 1:**
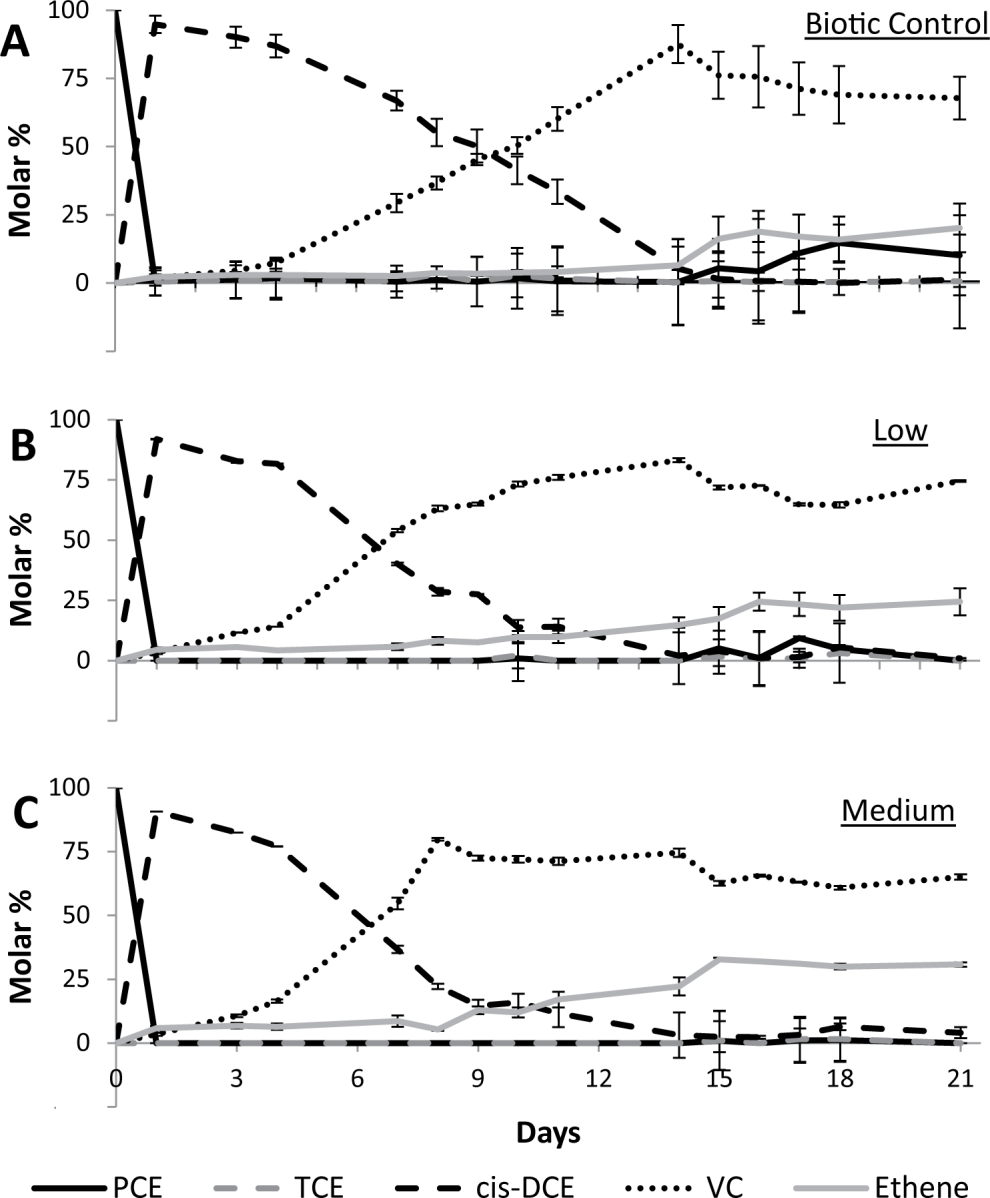
Molar percentage of chlorinated solvent PCE and degradation products TCE, *cis*-DCE, VC, and ethene. Results are given for (A) the biotic control, (B) low (25 μmol) permanganate treatment, and (C) medium (50 μmol) permanganate treatment. As the absolute mass increased over the course of the experiment due to PCE spiking (see Table 1), values are given as the molar percentage of all chlorinated compounds and ethene present at any time point. Headspace analysis of PCE, TCE, *cis*-DCE, VC, and ethene concentrations was always performed one day after each PCE spiking and nearly always in triplicate. the chemical analyses were shuffled between replicate microcosms of each treatment. Hence, the chemical results presented here are a conglomeration of chemical data from in total 9 microcosms.

During six doses of permanganate (three cycles of two doses per cycle) over the course of 21 days, OHR was not severely interrupted in microcosms receiving low and medium permanganate treatments of 25 μmol or 50 μmol (Fig 1). Rather, VC production and dechlorination rates were higher in microcosms receiving mild permanganate treatments as compared to the biotic control (Fig 1). For example, on day 8 more than twice as much VC had been produced in microcosms receiving medium permanganate doses, as compared to the biotic control (80% VC versus 37% VC, respectively). In the biotic control, the 80% threshold was not crossed until day 14, a delay of over a week compared to the medium permanganate treatment (Fig 1). Similarly, higher final ethene production was observed in permanganate treated microcosms as compared to the biotic control (31% in medium microcosms versus 20% in the biotic control on day 21). Additionally, PCE and TCE degradation in the first day after spiking was higher in microcosms receiving low or medium permanganate treatments (S1 Fig). In the biotic control microcosms, residual PCE or TCE were often observed the day after a PCE spike (Table 1 and Fig 1), with PCE spiked on days 16 and 17 remaining in the system until the end of incubation on day 21 (Fig 1A). In contrast, in microcosms treated with medium permanganate doses complete conversion of spiked PCE to *cis*-DCE occurred within 1 day after spiking in nearly all instances (Fig 1C), yielding consistent degradation rates (S1C Fig). These results indicate that chemical oxidation does not disrupt OHR; rather, mild treatments appear to slightly stimulate dechlorination.

**Fig 2:**
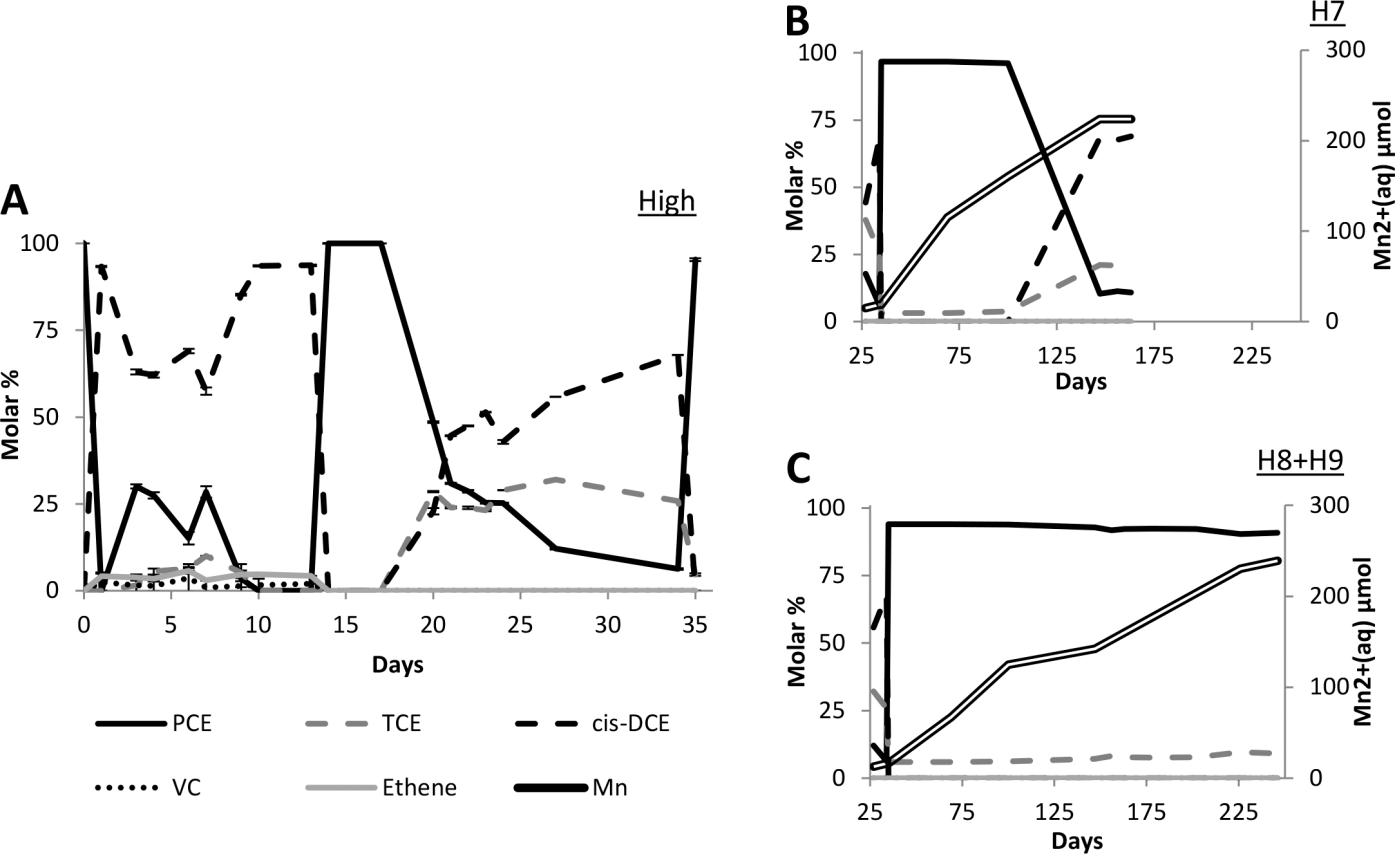
Molar percentage of chlorinated solvent PCE and degradation products TCE, *cis*-DCE, VC, and ethene and Mn^2+^. Results given for high (100 μmol) permanganate treatment, as described in Fig 1. (A) Days 1–33, during which the most PCE spiking and degradation occurred, are given in more detail. (B and C) Results from extended incubation period including Mn^2+^ concentrations. Results are split for H7 (B) and H8+H9 (C) to show difference in regeneration in degradation between these microcosms.

In contrast to the more mild permanganate treatments, significant disruption of OHR activity was observed in microcosms receiving high (100 μmol) permanganate doses (Fig 2). Following the first treatment on day 0, *cis*-DCE production in the high permanganate microcosms (93%; Fig 2) was similar to that of all other treatments (91–95%; Fig 1A–1C). However, following the second PCE spike on day 3, dehalogenation to *cis*-DCE required a week. Following a second permanganate treatment on day 13, dehalogenation was more significantly disrupted (Fig 2). Regeneration of PCE dechlorination coupled to TCE and *cis*-DCE accumulation resumed 4 days after the second permanganate addition on day 13. A third permanganate dose on day 33 severely disrupted OHR. Therefore, OHR activity was monitored over an extended period to determine if regeneration of PCE dechlorination could be observed. While some regeneration was measured on day 147 in H7, no dechlorination was observed in H8 and H9. Throughout the entire experiment, no VC or ethene production was observed at any point in microcosms receiving high permanganate treatments (Fig 2). Overall these results indicate that stronger chemical oxidant treatments can severely disrupt dechlorination.

Although OHR was disrupted in microcosms receiving high permanganate doses, chemical analyses of dissolved Mn indicated an active microbial community (Fig 2). Mn oxides, the product of permanganate reaction, can be utilized as an electron acceptor in dissimilatory Mn reduction to produce Mn^2+^. Aqueous Mn^2+^ concentrations steadily increased with prolonged incubation (Fig 2), indicating an actively respiring microbial community. However, as dechlorination of PCE was only observed in H7, Mn reduction was not the only factor limiting the recovery of OHR.

### 3.2 Influence of permanganate treatment on OHRB and rdh gene abundance

To understand the impact of chemical oxidation on microbial populations associated with OHR, the abundance of a variety of relevant OHRB and *rdh* genes was determined with qPCR. Total bacteria, total archaea, *D*. *mccartyi*, *Geobacter*, *Desulfitobacterium*, and *Sulfurospirillum* were measured based on their 16S rRNA gene sequence, and primers for specific *rdh* were used to detect *tceA*, *bvcA*, and *vcrA*, encoding TCE and two VC reductive dehalogenases in *D*. *mccartyi*, respectively (S1 Table and S2 Fig). In biotic controls and microcosms receiving low or medium permanganate treatments (25 or 50 μmol), an increase in the absolute abundance of total bacteria, *D*. *mccartyi*, and *Geobacter* was observed compared to the initial measurement on day 0, indicating their further growth over the course of the experiment (S2 Fig). *Dehalobacter* was not detected in any microcosms, independent of permanganate treatment.

**Fig 3:**
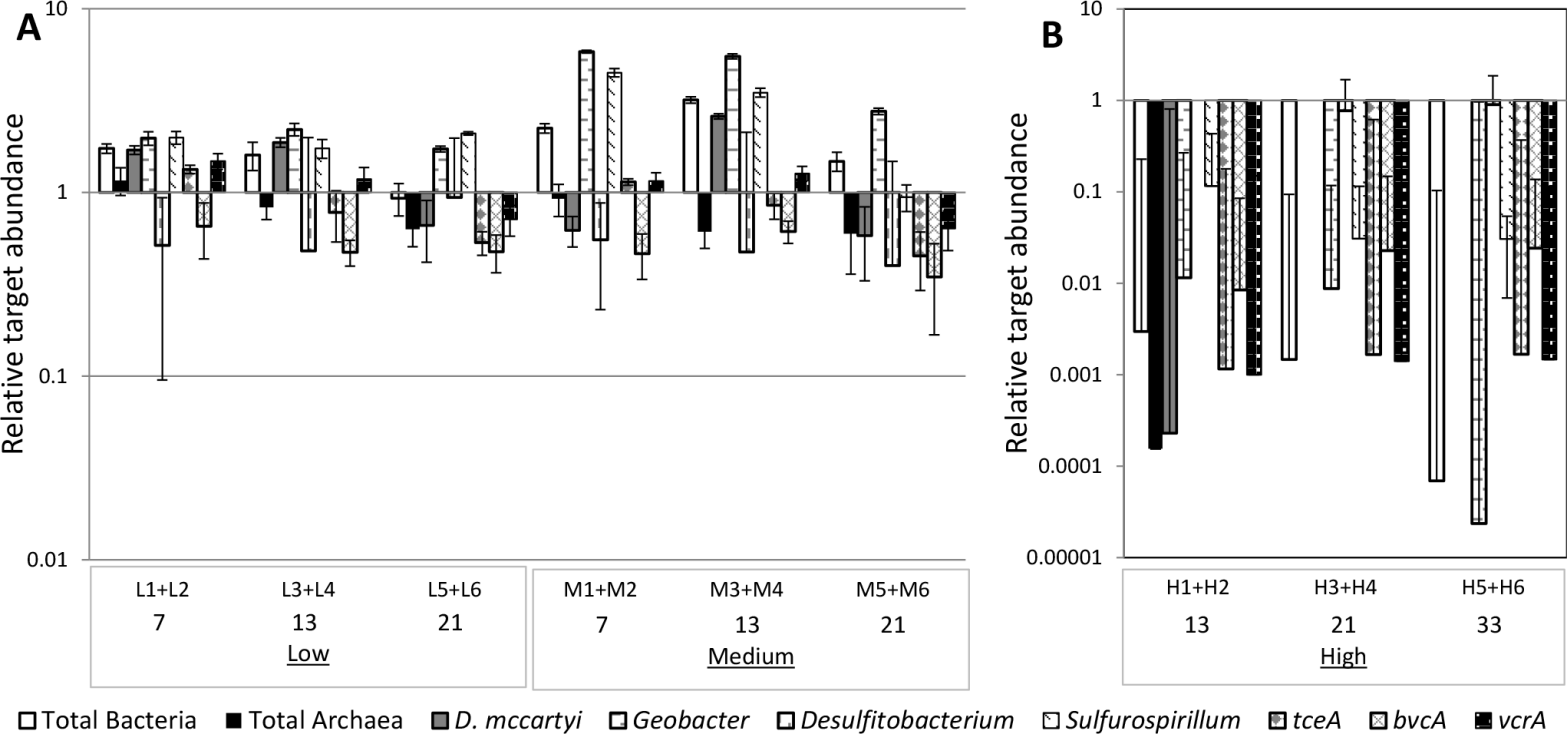
Relative target abundance of OHRB (based on 16S rRNA gene) and *rdh* genes measured by qPCR. Values indicate the enrichment or reduction of a target relative to that measured in the biotic control. Data is averaged between duplicate microcosms for each time point prior to comparison with the biotic control. Error bars are the propagation of standard deviation in triplicate assays, averaging of duplicates, and calculation of relative abundance. (A) Results in low or medium permanganate treated microcosms are compared to results from the biotic control at the same time point. (B) Results from the high permanganate microcosms were compared to the biotic control on day 13 (H1+H2) and day 21 (H3+H4, H5+H6). H7, H8, and H9 are not included, as biotic controls are not available for these time points. Note that different concentration scales are used for Y axes of (A) and (B) panels.

To account for natural shifts in target gene abundance during the course of the experiment, the abundance of each target gene observed in microcosms receiving permanganate was normalized to the corresponding target gene in the biotic controls at each time point (Fig 3). Overall higher total bacteria and *Geobacter* normalized abundances were observed in microcosms receiving low and medium permanganate treatments. In the case of *Geobacter*, a well-described Mn reducer [38], 3–6 times higher abundances were measured on days 7, 13, and 21 in microcosms receiving medium permanganate treatment than in the biotic control (Fig 3A). During the first two weeks, a slight enrichment of *D*. *mccartyi* and *vcrA* was detected in microcosms receiving low or medium permanganate treatments. However, at the last sampling time point on day 21, the abundances of both targets were lower than those of the biotic control. *Desulfitobacterium*, *bvcA*, and *tceA* were consistently lower in permanganate treated microcosms than in the biotic controls (Fig 3A). In the case of *bvcA* and *tceA*, this result was particularly noteworthy considering the higher PCE degradation rate observed in permanganate treated microcosms (S1 Fig).

In contrast, the abundance of all targets decreased in microcosms receiving high (100 μmol) permanganate doses (S2 Fig), showing the detrimental effect of stronger chemical oxidant treatments on the microbial community. In microcosms H3-H6, neither archaea nor *D*. *mccartyi* were detected on days 21 and 33, and *Sulfurospirillum* was only detected in one of these four microcosms (H5; S2 Fig). Additionally, high permanganate treatment caused a 2–4 orders of magnitude decrease in the normalized abundance of all detected targets (Fig 3B). With longer incubation periods, regeneration was observed for many targets to pre-treatment levels (S2 Fig). Notably, *D*. *mccartyi* was only detected in H7 and *Geobacter* was 3–4 orders of magnitude lower relative to initial samples (S2 Fig). The latter is notable considering this genus’s capacity for dissimilatory Mn reduction. Together these results indicate that stronger oxidation treatments severely disrupt the microbial community, which, even upon longer incubation periods, could not be completely restored.

### 3.3 Microbial community diversity and structure

In order to understand the influence of chemical oxidation on microbial community dynamics, PCR amplified partial 16S rRNA gene fragments were sequenced in samples from the different treatments and time points (Table 1). After filtering and trimming, between 269 and 24380 (average of 10086 ± 4113) high quality sequences were found per sample and clustered into 26–138 operational taxonomic units (OTUs; average 73 ± 23) per sample (Table 2). Although DNA sampling was always performed a number of days after permanganate treatment once regeneration of OHR was observed (Table 1), fewer sequences were obtained in samples receiving high permanganate treatment (Table 2). This is most likely due to the direct impact of oxidation on the microbial community. While Mn oxides could theoretically influence DNA extraction efficiency, previous studies on Mn oxide nodules did not indicate this [39, 40]. Regardless, due to the large variation in numbers of sequences obtained for each sample, alpha-diversity could not be properly assessed in this data set.

**Table 2:** Summary of treatments and timing. Samples were taken at start of experiment (“S”), and during treatment as biotic control (“B”), and receiving low (“L”), medium (“M”), or high (“H”) permanganate doses. Treatment times, cumulative permanganate (PM) and number of permanganate doses are used as model inputs for Table 3 and Fig 6 and S3 Fig. Number of sequences and operational taxonomic units (OTUs) obtained for each sample are given.

Sample Code	Treatment	Incubation Time(days)	Cumulative PM(μmol)	Doses PM	Sequences	OTUs
S1	Start	0	0	0	6689	80
S2	(Initial Samples)	0	0	0	8111	103
B1		7	0	0	10585	97
B2		7	0	0	7957	81
B3	Biotic Control	13	0	0	8311	68
B4	(No PM)	13	0	0	10247	60
B5		21	0	0	6791	54
B6		21	0	0	10059	77
L1		7	50	2	9451	67
L2		7	50	2	9194	73
L3	Low	13	100	4	9837	71
L4	(25 μmol PM)	13	100	4	11578	71
L5		21	150	6	10678	67
L6		21	150	6	11513	77
M1		7	100	2	11831	93
M2		7	100	2	10311	81
M3	Medium	13	200	4	12062	97
M4	(50 μmol PM)	13	200	4	10763	94
M5		21	300	6	9147	62
M6		21	300	6	9130	58
H1		13	100	1	9059	70
H2		13	100	1	24380	110
H3		21	200	2	10190	65
H4	High	21	200	2	7875	51
H5	(100 μmol PM)	33	200	2	2788	138
H6		33	200	2	269	26
H7		163	300	3	14507	40
H8		246	300	3	13126	60
H9		246	300	3	16056	37

**Fig 4:**
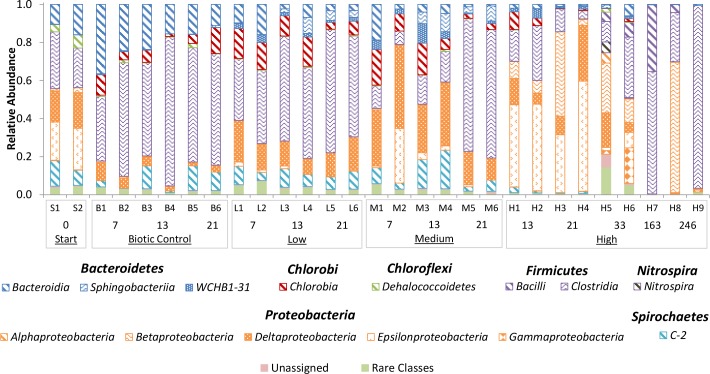
Relative abundance of dominant bacteria phyla and classes. Phyla (given by different colors) and classes (given by pattern) are based on partial 16S rRNA gene sequences obtained by pyrosequencing of barcoded PCR products. Phyla and classes are presented with at least a 0.05 abundance in any sample. Treatments and sample numbers are as given in Table 2. The length of incubation is given in days below the treatment labels.

**Fig 5:**
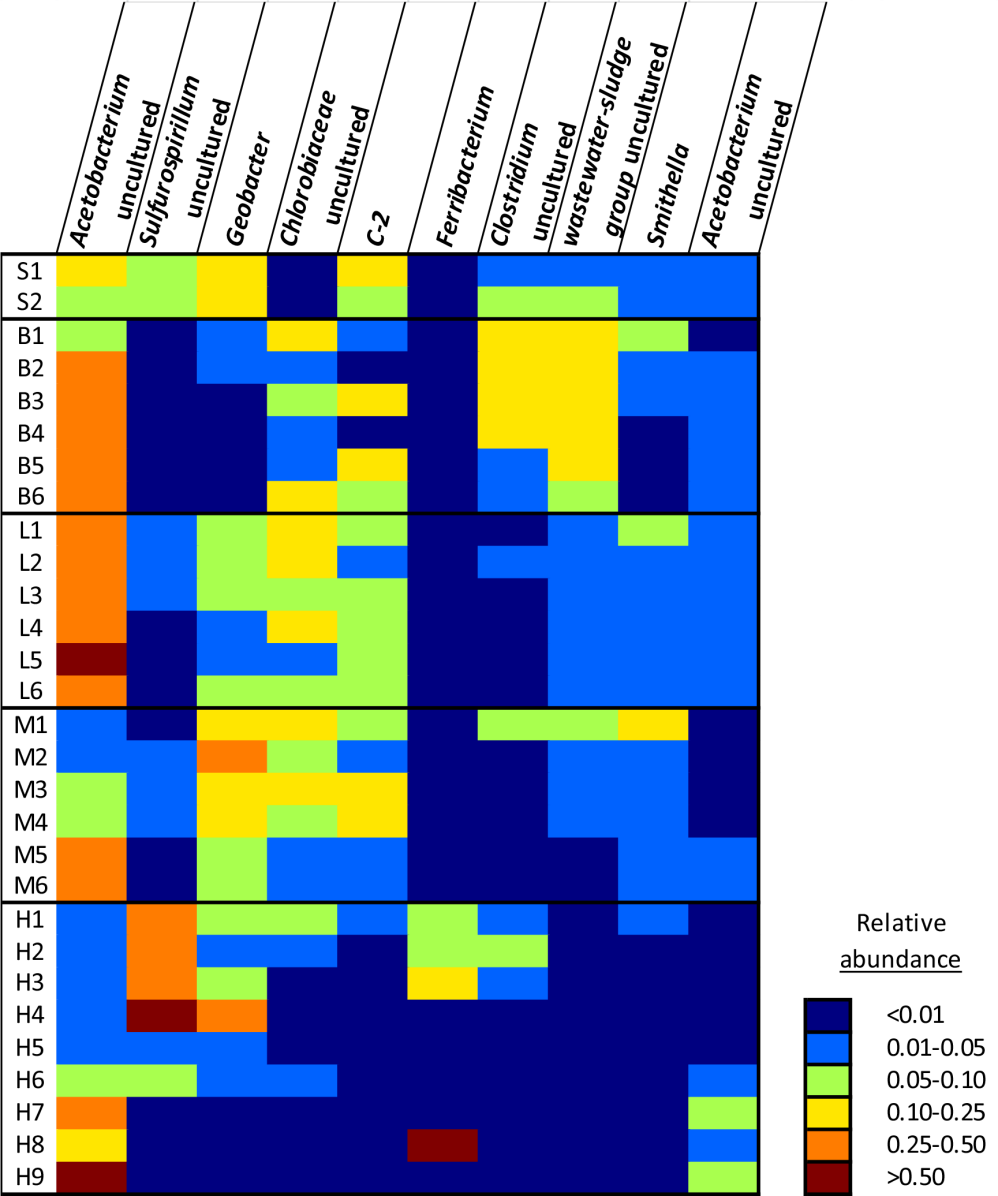
Heatmap of 10 most abundant OTUs in data set. Sample labels as in Table 2 and relative abundances are given as fraction of 1.

OTUs were classified into 31 bacterial phyla, with 98.3% of the OTUs belonging to 7 major phyla i.e. *Bacteriodetes*, *Chlorobi*, *Chloroflexi*, *Firmicutes*, *Nitrospirae*, *Proteobacteria*, and *Spirochaetes* (Fig 4). The most abundant OTUs were found to belong to the genus *Acetobacterium* in the class *Clostridia* (*Firmicutes*), the genus *Sulfurospirillum* in the class *Epsilonproteobacteria*, and *Geobacter* of the class *Deltaproteobacteria* (Fig 5). *Clostridia* were prevalent in many samples, with relative abundances up to 96% (H9; Fig 4). Overall, good correspondence was seen between duplicate microcosms, although high permanganate treatments (H3+H4, H5+H6) and longer incubation times (H8+H9) seemed to cause more divergence between the microbial community in replicate microcosms.

**Fig 6:**
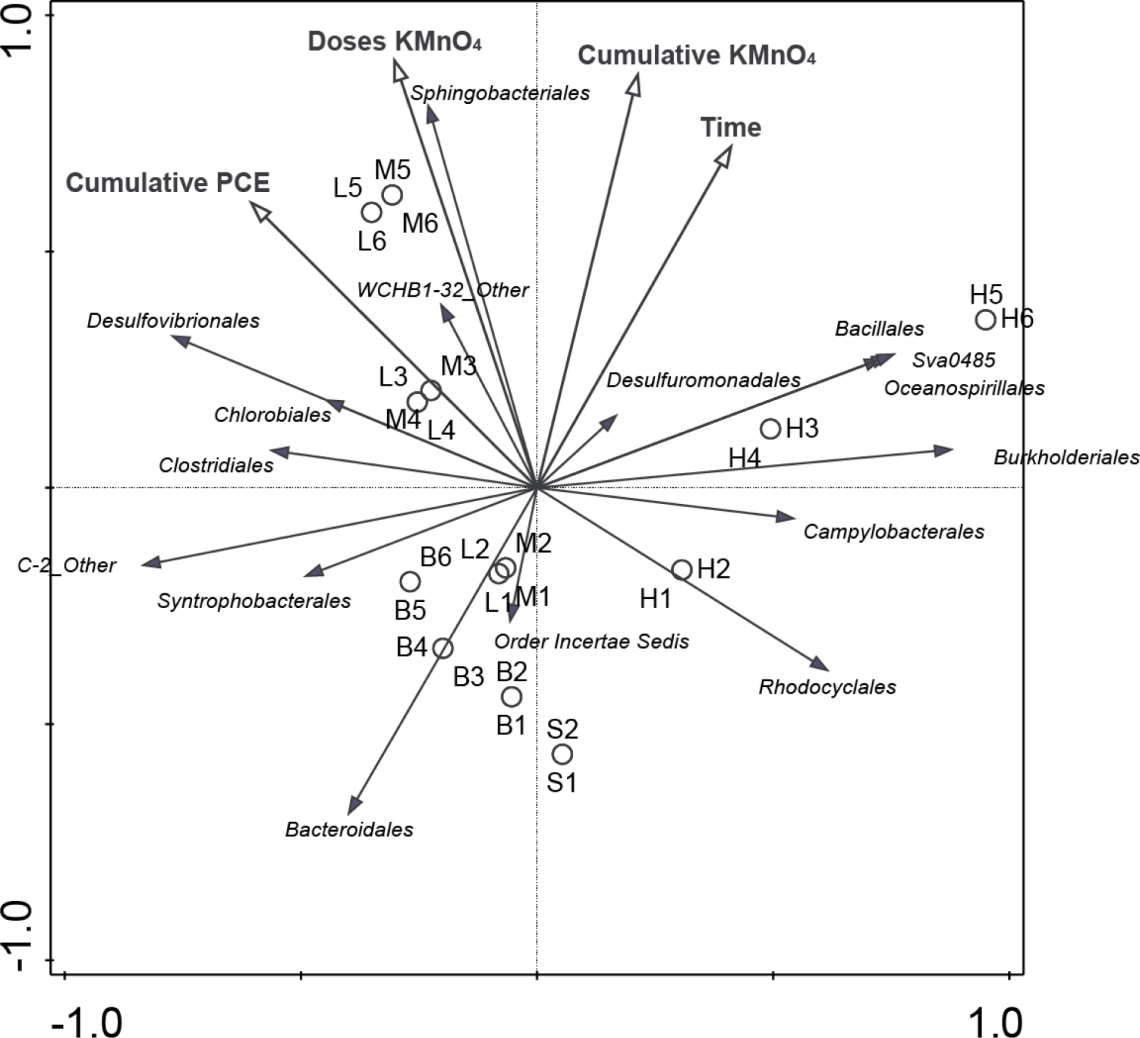
Redundancy Analysis Triplot showing relationship between microbial community composition at order level and treatments. Treatment variables are given as open arrows and are described in Table 2. Closed arrows represent orders. Orders were included with a relative abundance of at least 0.05 in any sample. Arrow length gives the variance that can be explained by a particular treatment parameter. Perpendicular distance reflects association, with smaller distances indicating a larger association. H7, H8 and H9 are left out of this plot because the long incubation times skew the scales, thus compressing the rest of the plot. A Triplot with all samples in this study is given in S3 Fig.

In order to understand the impact of chemical treatment on microbial diversity, statistical models were used to determine the interaction among a number of variables and community structure. Chemical oxidation was assessed in terms of number of doses as well as cumulative permanganate added (Table 2), thereby accounting for the potentially different impact of one large addition versus a number of smaller doses of permanganate. Incubation time was examined, however, for some redundancy analyses H7-H9 were analyzed separately, as the long incubation times skewed the scales and interpretation of the results (Fig 6 and S3 Fig). Finally, PCE dosing was included, however, as PCE spiking was only performed when degradation occurred, this should not be considered a completely independent variable.

Redundancy analysis triplots showed a shift in the community upon incubation (Fig 6). Biotic control microcosms developed away from the starting samples (“S”) over time. A strong association could be observed between biotic control microcosms and the order *Bacteroidales* (Fig 6), which is supported by the negative association between cumulative permanganate addition and time and this genus (Table 3). In biotic control microcosms, the order *Bacteroidales* was dominated by the *wastewater sludge group* of the family *Rikenellaceae*, which was notably absent in permanganate treated microcosms (Fig 5). Finally, while the class *Dehalococcoidetes*, dominated by the genus *Dehalococcoides*, was associated with the biotic control, a negative association with incubation time could be observed (Fig 6, Table 3).

**Table 3:** Association between treatment variables and microbial community composition at order level. Treatment variables are from Table 2. Orders were included with relative abundances above 0.05 in any sample. Significant associations are given in bold (*P*-value < 0.01).

Class	Order	Cumulative KMnO_4_	Doses KMnO_4_	Time	Treatment Type
*Bacilli*	*Bacillales*	**<0.001**	**<0.001**	**<0.001**	1.000
*Bacteroidia*	*Bacteroidales*	**<0.001**	0.593	0.023	0.376
*Betaproteobacteria*	*Burkholderiales*	0.017	0.098	0.633	0.125
*Betaproteobacteria*	*Rhodocyclales*	0.637	0.298	0.484	0.664
*C-2*	(Unclassified)	0.091	**<0.001**	0.470	**<0.001**
*Chlorobia*	*Chlorobiales*	**0.004**	0.059	0.249	0.072
*Clostridia*	*Clostridiales*	**0.002**	0.355	0.131	0.448
*Dehalococcoidetes*	*Order Incertae Sedis*	**0.005**	0.986	**<0.001**	0.718
*Deltaproteobacteria*	*Desulfovibrionales*	0.025	0.012	0.783	0.061
*Deltaproteobacteria*	*Desulfuromonadales*	0.035	0.285	0.012	0.086
*Deltaproteobacteria*	*Sva0485*	0.384	0.576	0.084	1.000
*Deltaproteobacteria*	*Syntrophobacterales*	**0.008**	0.032	0.054	0.119
*Epsilonproteobacteria*	*Campylobacterales*	**0.002**	**<0.001**	**0.002**	0.055
*Gammaproteobacteria*	*Oceanospirillales*	0.523	0.671	0.159	1.000
*Sphingobacteriia*	*Sphingobacteriales*	0.033	**<0.001**	0.999	0.348
*WCHB1-32*	(Unclassified)	0.011	0.016	0.185	**0.005**

Microcosms receiving low or medium permanganate treatments showed close clustering while deviating from the starting sample and biotic control (Fig 6). The variables time, cumulative permanganate addition, and especially permanganate doses showed a stronger association with these treatments. Particularly correlated with low and medium permanganate samples were *Sphingobacteria*, as shown both in triplot (Fig 6) and modelling (Table 3). This order was dominated by unclassified *WCHB1-69* clone, which has been observed in a number of dechlorinating enrichment cultures [41, 42]. Another unclassified clone of the phylum *Bacteroidetes*, *WCHB1-32*, previously observed in a debrominating culture [43], was also associated with low and medium permanganate treatments. Finally, the order *Clostridales*, dominated by *Acetobacterium*, plots between biotic control and low and medium permanganate dose microcosms (Fig 6). The prevalence of a number of OTUs classified as *Acetobacterium*, an important fermenter identified in a variety of dechlorinating enrichment cultures [11, 44–46] was observed in all three treatments, however at very different relative abundances (Fig 5).

Analysis of the microbial diversity in the microcosms treated with high permanganate doses indicated divergence from the communities observed in low and medium permanganate treatments (Fig 6). The orders *Bacillales*, *Burkholderiales*, *Campylobacterales* and *Oceanospirillales*, as well as the uncultured *Deltaproteobacteria Sva0485* were especially associated with samples receiving high permanganate doses. *Campylobacterales* were dominated by the genus *Sulfurospirillum*, with an uncultured species being the most abundant OTU in samples H1-H4 (Fig 5). *Sulfurospirillum* is known to perform OHR as well as dissimilatory Mn reduction [38, 47]. In addition to *Sulfurospirillum*, *Geobacter* of the order *Desulfuromonadales*, *Campylobacterales* and *Oceanospirillales* were other Mn reducers found to be associated with high permanganate treatment ([38, 48]; Figs 5 and 6). The OTU categorized as *Acetobacterium*, which was found to be highly abundant in other microcosms was found in very low abundance in H1-H6 following high permanganate doses.

## Discussion

Permanganate treatment induced changes in dechlorination rates (Figs 1 and 2 and S1 Fig), OHRB and *rdh* gene abundance (Fig 3 and S2 Fig), and microbial community structure (Figs 4–6), as compared to the biotic control. A similar response to chemical oxidation was observed in microcosms receiving low or medium permanganate doses (25 or 50 μmol) which was in sharp contrast to the trend observed upon application of high (100 μmol) permanganate doses. Overall, results indicate a strong dose-dependency: single application of a high quantity of permanganate can have a severe detrimental effect on OHR, while repeated lower doses yielding the same cumulative quantity of permanganate can have a slightly stimulatory effect.

Permanganate treatment can be detrimental by causing direct cell damage, as seen in the decrease in community size observed following treatment with a variety of chemical oxidants [22, 26, 49]. Additionally, interruption of OHR due to a temporary increase in the redox potential may also be expected. However, the accumulation of Mn oxides seemed to stimulate OHR, as described below. Analysis of microbial community composition in each of the treatments indicated that a balance between detrimental oxidative stress and stimulation with Mn oxides is necessary in order to ensure regeneration of OHR activity following chemical oxidation. Specifically, results indicated that knowledge on a combination of the dynamics of OHRB, Mn reducing bacteria, and non-dechlorinating OHR-supporting community members are key to understanding and predicting the result of permanganate treatment on OHR.

In microcosms receiving high permanganate doses, significant reduction in OHR rates and interruption of dechlorination beyond *cis*-DCE was observed. The sensitivity of OHRB towards oxidative stress has been published previously [50, 51]. A comprehensive study by Amos et al. showed that oxidative stress (up to 30 days of oxygen exposure) disrupts OHR and irreversibly impedes degradation of VC to ethene [50], similar to the stalling at *cis*-DCE observed in our study.

However, it appears that the mechanism of interruption by permanganate treatment is more detrimental to OHRB than that for oxygen. In the study of Amos et al., qPCR analysis showed a less than one order of magnitude drop in the abundance of the *D*. *mccartyi* 16S rRNA gene and the *rdh* genes *tceA* and *vcrA* upon oxygen exposure, yielding the conclusion that these biomarkers were indicative of presence but were not predictive of cell viability. In contrast, in our high permanganate treatments, a 2 order of magnitude reduction in overall community size and the absence of a number of OHRB 16S rRNA targets was measured (Fig 3 and S2 Fig). These results suggest that permanganate concentrations were high enough to inflict direct cell damage, rather than merely inhibiting OHR by oxidative stress. While the overall community size did regenerate during prolonged incubation (S2C Fig, H7-9), the absence of *D*. *mccartyi* as the only known microbes capable of complete PCE dechlorination is particularly challenging. It has been shown that at low abundances, *D*. *mccartyi* can be outcompeted for electron donor and growth substrates [16], implying that permanganate-induced reduction in abundance may not be reversed. These results indicate that increasing the oxidant dose beyond a certain threshold quantity for *D*. *mccartyi* may irreversibly knockout this organism from OHR consortia, which may lead to the accumulation of more toxic dechlorination intermediates such as *cis*-DCE and VC.

On the other hand, low and medium permanganate treatments were neither detrimental to OHRB and *rdh* abundance nor did these mild treatments interrupt OHR (Fig 3 and S1 Fig). Rather, higher dechlorination rates (Fig 1) and relative abundance of biomarkers (Fig 2) were observed as compared to the biotic control. The observed increase in PCE dechlorination relative to the biotic control can to some extent be explained by the enrichment of a number of target guilds able to utilize permanganate-deposited Mn oxides, in addition to chlorinated ethenes, as terminal electron acceptors. The enrichment of Mn reducing OHRB such as *Geobacter* and *Sulfurospirillum* in low and medium permanganate treatments, as compared to the biotic control (Fig 3), may be attributed to their diverse metabolic capacity [47, 52] and, in the case of *Sulfurospirillum*, apparent resilience to oxidative stress [53]. In addition to the energetic advantage afforded by dissimilatory Mn reduction [54, 55], these genera also have the competitive advantage of being able to switch to chlorinated ethenes as an electron acceptor once Mn oxides have been consumed.

In addition to supporting the higher dechlorination rates observed in low and medium permanganate treatments, Mn reducers contributed to the attenuation of Mn oxides following chemical oxidation. Members of the orders *Campylobacterales*, *Oceanospirillales*, and *Rhodocyclales*, found in especially high permanganate treatment microcosms (Fig 6), may also have played a role in reduction of Mn oxides. Stable isotope probing of enrichments from three different Mn oxide rich sediments in Sweden, Norway, and Korea identified *Arcobacter* of the order *Campylobacterales* and an unclassified clone in the order *Oceanospirillales* as likely Mn-reducers [48]. Additionally, an OTU classified as the Fe(III) reducer *Ferribacterium* of the order *Rhodocyclales* was particularly prevalent in a number of high permanganate treatment microcosms (Fig 5; [56]). While Mn reduction has not been specifically demonstrated by this genus, the overlapping metabolic capabilities of dissimilatory metal reduction suggest that this OTU may be involved in Mn reduction. It is noteworthy that different Mn reducers were enriched in the high permanganate treated microcosms, where *Geobacter* was not enriched, as compared to the low and medium permanganate treatments. The lower abundances of *Geobacter* in high dose microcosms indicate that this genera may have been particularly susceptible to the detrimental impact of oxidative stress, in line with research showing that this genera lacks homologs of a variety of oxidative stress detoxification enzymes [57].

A number of non-dechlorinating community members identified in permanganate treated microcosms potentially supported OHR through a variety of syntrophic interactions, while profiting from dechlorination of toxic chlorinated compounds. Previous metagenomic analysis of three dechlorinating enrichment cultures identified a number of organisms potentially supportive of *D*. *mccartyi* with specific metabolic pathways, including corrinoid synthesis, methionine synthesis, oxygen scavenging, and electron donor metabolism [11, 12]. That work identified the role of fermenters associated with *Firmicutes* within the order *Clostridiales*, specifically members of the genus *Acetobacterium* and the family *Clostridiaceae*, as key producers of vitamins and co-factors. In our study, members of the order *Clostridiales* appeared to be correlated with low and medium permanganate treatments and strongly negatively associated with high permanganate treatments (Fig 6). Additionally, an OTU classified as *Acetobacterium* was notably absent in microcosms treated with high permanganate doses just after treatment (H1-H6; Fig 5). While the class *Clostridia* as well as the specific OTU showing similarity to *Acetobacterium* did regenerate upon longer incubation periods (H7-H9; Figs 4 and 6), the low relative abundance of *Dehalococcoides* and perhaps other OHRB precluded regeneration of dechlorination, as suggested previously [16]. Together, these results appear to indicate that strong permanganate treatments are not only detrimental towards OHRB, but also to non-dechlorinating community members essential to the activity of OHRB within consortia.

Thus, analysis of the microbial community diversity in PCE biodegrading microcosms treated with permanganate at different doses gave an indication of mechanisms by which permanganate treatment can either stimulate or interrupt OHR. In low and medium permanganate treatments, specific Mn-reducing, dechlorinating OHRB such as *Geobacter* and *Sulfurospirillum* were enriched that are flexible in using a wider range of electron acceptors. Additionally, stimulated non-dechlorinating supportive guilds such as fermenters within *Clostridiales* and sulfate reducing *Deltaproteobacteria* (Fig 4) could further provide *Dehalococcoides* with essential co-factors, resulting in higher dechlorination rates than in the biotic control, as suggested in other studies [11]. In contrast, high permanganate treatments were strong enough to cause direct cell damage to OHRB as well as equally important non-dechlorinating community members. These results indicate that stronger oxidation treatments may irreversibly knockout *D*. *mccartyi* from OHR consortia, due to its own strict metabolism and decrease in the abundance of OHR-supporting guilds. In practical applications, the absence of *D*. *mccartyi* may lead to the accumulation of more toxic dechlorination intermediates such as *cis*-DCE and VC, which pose a higher risk at contaminated locations.

Overall, the results presented here indicate that mild permanganate treatments can have a stimulatory effect on PCE dechlorination, while stronger oxidant loads can completely and irreversibly disrupt OHR. Additionally, our results are the first to report that a number of OHRB appear to be enriched by permanganate treatment and may transiently utilize Mn oxides as an alternative electron acceptor. Enrichment upon permanganate treatment was also indicated for key supportive organisms such as genera within the class *Clostridiales* and *Deltaproteobacteria*, whose consistent abundance throughout permanganate treatment may be indicative of whether or not chemical oxidation will have a stimulatory or detrimental impact on OHR. These important results shed new light on the relationship between OHRB and Mn reducing bacteria in the environment, knowledge which can be exploited in applied field situations to be able to better predict the resiliency of OHR towards permanganate treatment, develop new biomolecular tools targeting organisms key to OHR following chemical oxidation, and eventually design effective and efficient chemical and biological remediation scenarios for full scale field application.

## Supporting Information

S1 Fig PCE and TCE degradation rates. Rates given for PCE (black) and TCE (grey) in the first day after spiking in biotic control (A), low (25 μmol) permanganate (B), and medium (50 μmol) permanganate treatment (C) microcosms. The x-axis indicates the period for which degradation was measured (for example, spiking on day 9 and measurement on day 10). Rates of 0.86 μmol/day indicate full degradation of the 0.86 μmol PCE spike within one day.
(PDF)

S2 Fig OHRB and *rdh* gene abundance for individual microcosms. Individual microcosm numbers are given in Table 2. Error bars are standard deviation of triplicate assays.
(PDF)

S3 Fig Redundancy Analysis Triplot showing relationship between microbial community composition at order level and treatments. Treatment variables are given as open arrows and are described in Table 2. Closed arrows represent orders. Orders were included with a relative abundance of at least 0.05 in any sample. Arrow length gives the variance that can be explained by a particular treatment parameter. Perpendicular distance reflects association, with smaller distances indicating a larger association.
(PDF)

S1 Table Overview of primers used for qPCR assays. (PDF)

S2 Table Relative abundances of bacterial orders. Orders with relative abundances of more than 0.05 in any sample were included in analyses in Table 3 and Fig 6.
(PDF)
