# Supplementary material for: Microbial Community Response of an Organohalide Respiring Enrichment Culture to Permanganate Oxidation
Source: PLoS One. 2015 Aug 5;10(8):e0134615. doi: 10.1371/journal.pone.0134615 (PMC4526698; doi:10.1371/journal.pone.0134615)
Supplement: S1 Fig — Rates given for PCE (black) and TCE (grey) in the first day after spiking in biotic control (A), low (25 μmol) permanganate (B), and medium (50 μmol) permanganate treatment (C) microcosms. The x-axis indicates the period for which degradation was measured (for example, spiking on day 9 and measurement on day 10). Rates of 0.86 μmol/day indicate full degradation of the 0.86 μmol PCE spike within one day. (PDF) [file pone.0134615.s001.pdf]

Microbial community response of an organohalide respiring enrichment culture to permanganate oxidation

Nora B. Sutton[1], Siavash Atashgahi[2], Edoardo Saccenti[3],Tim Grotenhuis[1], Hauke Smidt[2], and Huub H.M. Rijnaarts[1]

[1] Environmental Technology, Wageningen University, Wageningen, The Netherlands

[2] Laboratory of Microbiology, Wageningen University, Wageningen, The Netherlands

[3] Laboratory of Systems and Synthetic Biology, Wageningen University, Wageningen, The Netherlands

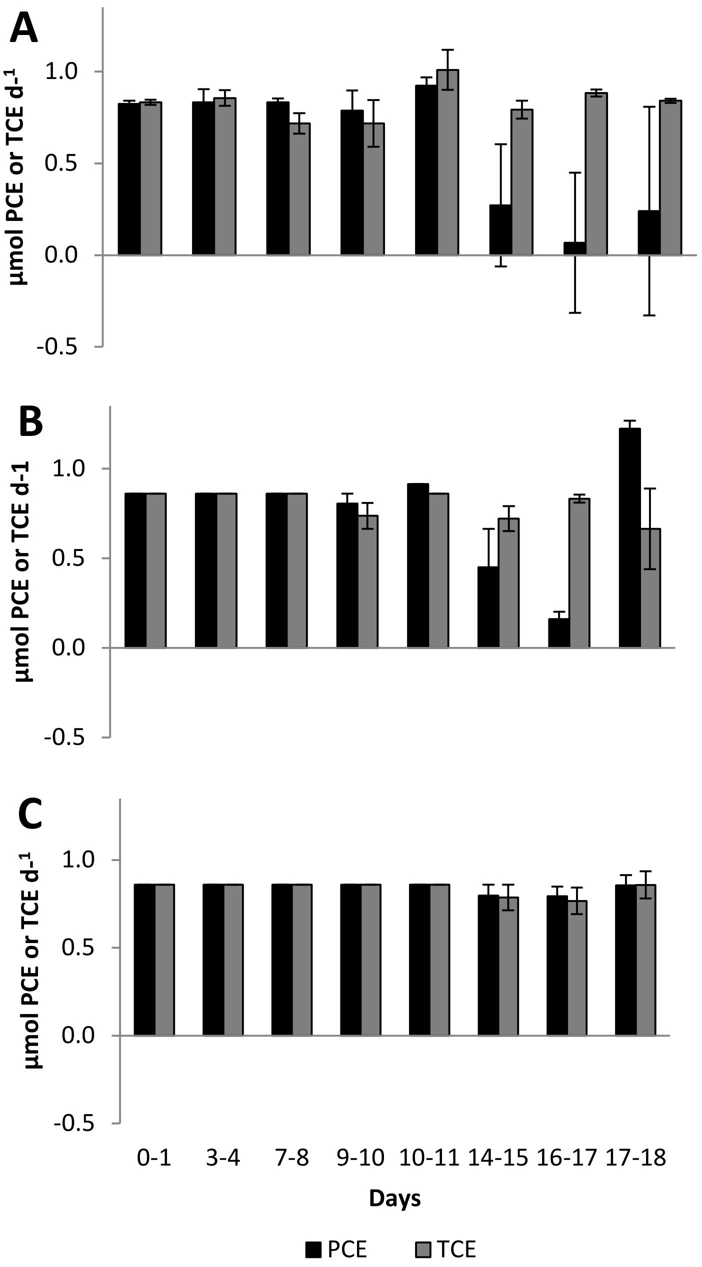

S1 Fig. PCE and TCE degradation rates. Rates given for PCE (black) and TCE (grey) in the first day after spiking in biotic control (A), low (25 µmol) permanganate (B), and medium (50 µmol) permanganate treatment (C) microcosms. The x-axis indicates the period for which degradation was measured (for example, spiking on day 9 and measurement on day 10). Rates of 0.86 µmol/day indicate full degradation of the 0.86 µmol PCE spike within one day.
