# Supplementary material for: Microbial Community Response of an Organohalide Respiring Enrichment Culture to Permanganate Oxidation
Source: PLoS One. 2015 Aug 5;10(8):e0134615. doi: 10.1371/journal.pone.0134615 (PMC4526698; doi:10.1371/journal.pone.0134615)
Supplement: S2 Fig — Individual microcosm numbers are given in Table 2. Error bars are standard deviation of triplicate assays. (PDF) [file pone.0134615.s002.pdf]

Microbial community response of an organohalide respiring enrichment culture to permanganate oxidation

Nora B. Sutton[1], Siavash Atashgahi[2], Edoardo Saccenti[3],Tim Grotenhuis[1], Hauke Smidt[2], and Huub H.M. Rijnaarts[1]

[1] Environmental Technology, Wageningen University, Wageningen, The Netherlands

[2] Laboratory of Microbiology, Wageningen University, Wageningen, The Netherlands

[3] Laboratory of Systems and Synthetic Biology, Wageningen University, Wageningen, The Netherlands

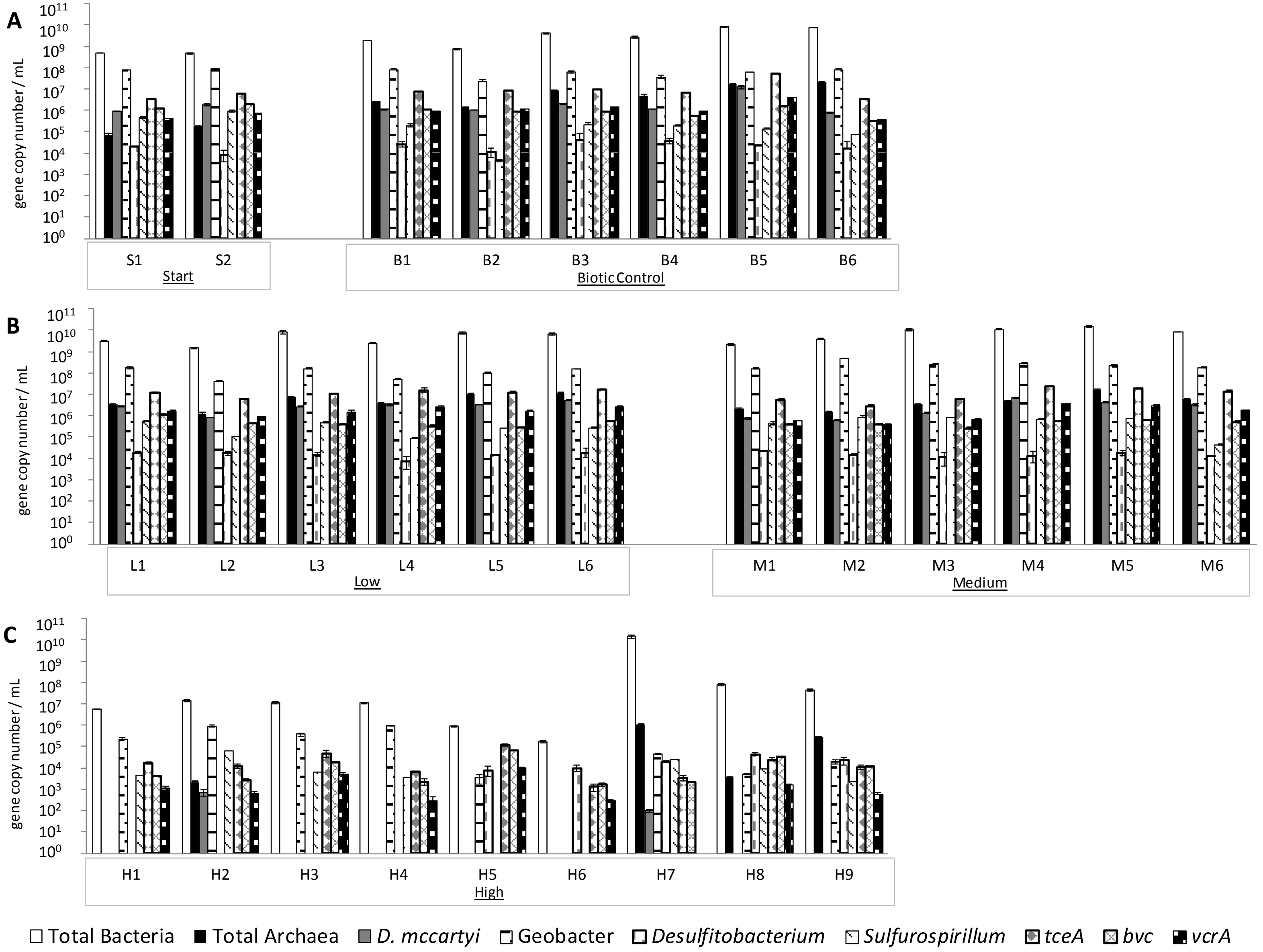

S2 Fig. OHRB and *rdh* gene abundance for individual microcosms. Individual microcosm numbers are given in Table 2. Error bars are standard deviation of triplicate assays.
