# Supplementary material for: Microbial Community Response of an Organohalide Respiring Enrichment Culture to Permanganate Oxidation
Source: PLoS One. 2015 Aug 5;10(8):e0134615. doi: 10.1371/journal.pone.0134615 (PMC4526698; doi:10.1371/journal.pone.0134615)
Supplement: S3 Fig — Treatment variables are given as open arrows and are described in Table 2. Closed arrows represent orders. Orders were included with a relative abundance of at least 0.05 in any sample. Arrow length gives the variance that can be explained by a particular treatment parameter. Perpendicular distance reflects association, with smaller distances indicating a larger association. (PDF) [file pone.0134615.s003.pdf]

Microbial community response of an organohalide respiring enrichment culture to permanganate oxidation

Nora B. Sutton[1], Siavash Atashgahi[2], Edoardo Saccenti[3],Tim Grotenhuis[1], Hauke Smidt[2], and Huub H.M. Rijnaarts[1]

[1] Environmental Technology, Wageningen University, Wageningen, The Netherlands

[2] Laboratory of Microbiology, Wageningen University, Wageningen, The Netherlands

[3] Laboratory of Systems and Synthetic Biology, Wageningen University, Wageningen, The Netherlands

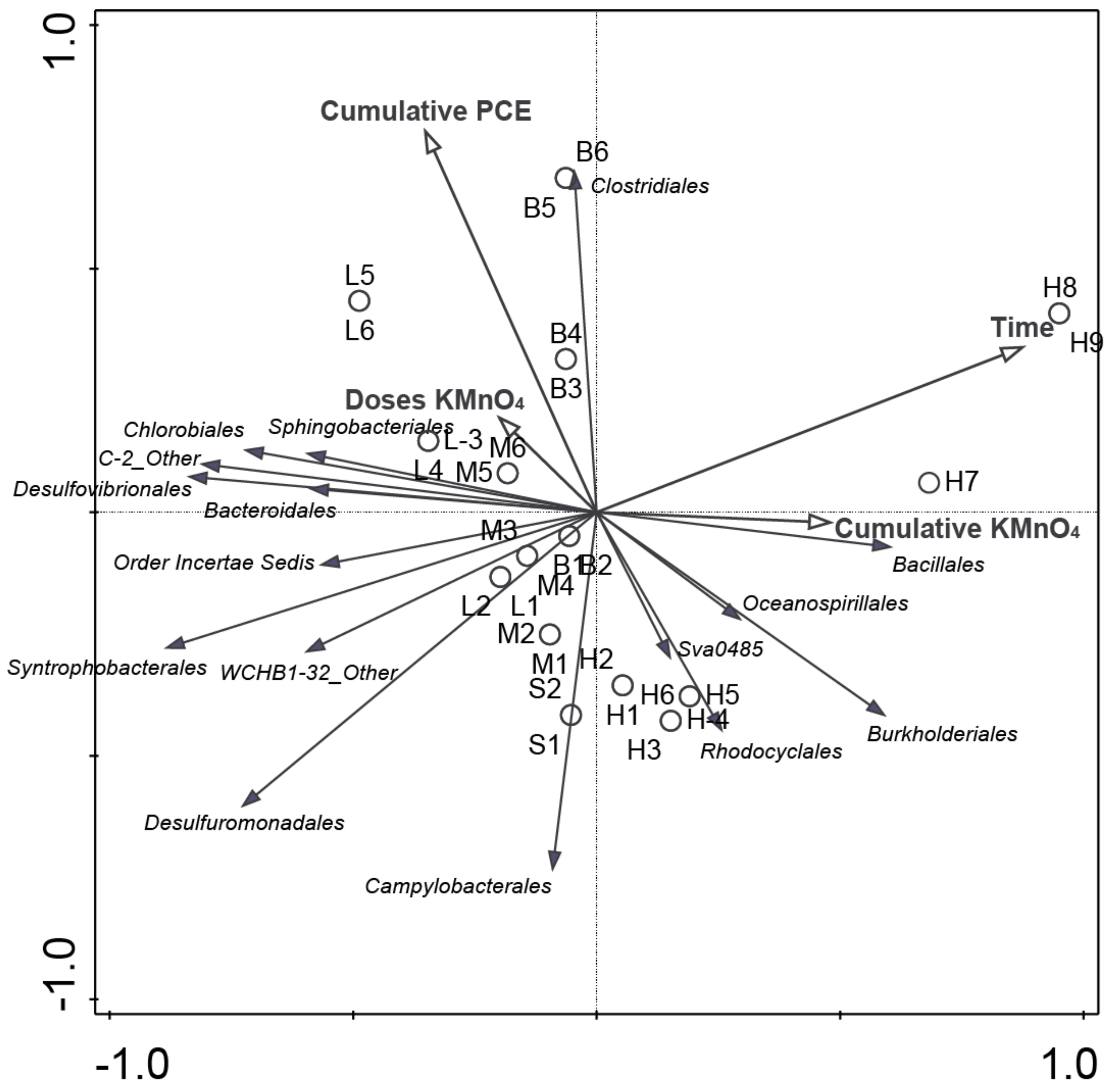

S3 Fig. Redundancy Analysis Triplot showing relationship between microbial community composition at order level and treatments. Treatment variables are given as open arrows and are described in Table 2. Closed arrows represent orders. Orders were included with a relative abundance of at least 0.05 in any sample. Arrow length gives the variance that can be explained by a particular treatment parameter. Perpendicular distance reflects association, with smaller distances indicating a larger association.
