# Supplementary material for: Microbial Community Response of an Organohalide Respiring Enrichment Culture to Permanganate Oxidation
Source: PLoS One. 2015 Aug 5;10(8):e0134615. doi: 10.1371/journal.pone.0134615 (PMC4526698; doi:10.1371/journal.pone.0134615)
Supplement: S1 Table — (PDF) [file pone.0134615.s004.pdf]

Microbial community response of an organohalide respiring enrichment culture to permanganate oxidation

Nora B. Sutton[1], Siavash Atashgahi[2], Edoardo Saccenti[3],Tim Grotenhuis[1], Hauke Smidt[2], and Huub H.M. Rijnaarts[1]

[1] Environmental Technology, Wageningen University, Wageningen, The Netherlands

[2] Laboratory of Microbiology, Wageningen University, Wageningen, The Netherlands

[3] Laboratory of Systems and Synthetic Biology, Wageningen University, Wageningen, The Netherlands

S1 Table. Overview of primers used for qPCR assays.

Primer	Sequence	Target 16S rRNA / gene	Reference
341F<br>534R	CCTACGGGAGGCAGCAG<br>ATTACCGCGGCTGCTGGC	All Bacteria	(1)
Arc787F<br>Arc1059R	ATTAG ATACC CSBGT AGTCC<br>GCCAT GCACC WCCTC T	Archaea	(2)
Dco728F<br>Dco944R	AAGGCGGTTTTCTAGGTTGTCAC<br>CTTCATGCATGTCAAAT	*Dehalococcoides mccartyi*	(3)
Dre441F<br>Dre645R	GTTAGGGAAGAACGGCATCTGT<br>CCTCTCCTGTCCTCAAGCCATA	*Dehalobacter*	(3)
Geo557F<br>Geo822R	GCGTGTAGGCGGTTTSTTAA<br>TACCCGCRACACCTAGTACT	*Geobacter*	(4)
Dsb406F<br>Dsb619R	GTACGACGAAGGCCTTCGGT<br>CCCAGGGTTGAGCCCTAGGT	*Desulfitobacterium*	(3)
Smul<br>Smul	GAGACACGGTCCAGACTCCTAC<br>CTCGACTTGATTTCCAGCCTAC	*Sulfurospirillum*	(Goris et al, unpublished data)
TceA1270F<br>TceA1336R	ATCCAGATTATGACCCTGGTGAA<br>GCGGCATATATTAGGGCATCTT	*tceA*	(5)
Bvc925F<br>Bvc1017R	AAAAGCACTTGGCTATCAAGGAC<br>CCAAAAGCACCACCAGGTC	*bvcA*	(6)
Vcr1022F<br>Vcr1093R	CGGGCGGATGCACTATTTT<br>GAATAGTCCGTGCCCTTCCTC	*vcrA*	(6)

References

1. Muyzer G, Dewaal EC, Uitterlinden AG. Profiling of complex microbial populations by denaturing gradient gel electrophoresis analysis of polymerase chain reaction amplified genes coding for 16S ribosomal RNA. Applied and Environmental Microbiology. 1993;59(3):695-700. PubMed PMID: WOS:A1993KQ12300007.
2. Yu Y, Lee C, Kim J, Hwang S. Group-specific primer and probe sets to detect methanogenic communities using quantitative real-time polymerase chain reaction. Biotechnol Bioeng. 2005;89(6):670-9. doi: 10.1002/bit.20347. PubMed PMID: WOS:000227247700006.
3. Smits THM, Devenoges C, Szynalski K, Maillard J, Holliger C. Development of a real-time PCR method for quantification of the three genera Dehalobacter, Dehalococcoides, and Desulfitobacterium in microbial communities. Journal of microbiological methods. 2004;57(3):369-78. doi: 10.1016/j.mimet.2004.02.003. PubMed PMID: WOS:000221631400006.
4. Azizian MF, Marshall IPG, Behrens S, Spormann AM, Semprini L. Comparison of lactate, formate, and propionate as hydrogen donors for the reductive dehalogenation of trichloroethene in a continuous-flow column. J Contam Hydrol. 2010;113(1-4):77-92. doi: 10.1016/j.jconhyd.2010.02.004. PubMed PMID: WOS:000276766000006.
5. Johnson DR, Lee PKH, Holmes VF, Alvarez-Cohen L. An internal reference technique for accurately quantifying specific mRNAs by real-time PCR with a application to the tceA reductive dehalogenase gene. Applied and Environmental Microbiology. 2005;71(7):3866-71. doi: 10.1128/aem.71.7.3866-3871.2005. PubMed PMID: WOS:000230445700060.
6. Ritalahti KM, Amos BK, Sung Y, Wu QZ, Koenigsberg SS, Loffler FE. Quantitative PCR targeting 16S rRNA and reductive dehalogenase genes simultaneously monitors multiple Dehalococcoides strains. Applied and Environmental Microbiology. 2006;72(4):2765-74. doi: 10.1128/aem.72.4.2765-27774.2006. PubMed PMID: WOS:000236749400060.
