# Supplementary material for: Microbial Community Response of an Organohalide Respiring Enrichment Culture to Permanganate Oxidation
Source: PLoS One. 2015 Aug 5;10(8):e0134615. doi: 10.1371/journal.pone.0134615 (PMC4526698; doi:10.1371/journal.pone.0134615)
Supplement: S2 Table — Orders with relative abundances of more than 0.05 in any sample were included in analyses in Table 3 and Fig 6. (PDF) [file pone.0134615.s005.pdf]

**Microbial community response of an organohalide respiring enrichment culture to permanganate oxidation**

Nora B. Sutton[1], Siavash Atashgahi[2], Edoardo Saccenti[3],Tim Grotenhuis[1], Hauke Smidt[2], and Huub H.M. Rijnaarts[1]

[1] Environmental Technology, Wageningen University, Wageningen, The Netherlands

[2] Laboratory of Microbiology, Wageningen University, Wageningen, The Netherlands

[3] Laboratory of Systems and Synthetic Biology, Wageningen University, Wageningen, The Netherlands

**S2 Table. Relative abundances of bacterial orders.** Orders with relative abundances of more than 0.05 in any sample were included in analyses in Table 3 and Fig. 6.

| | *Bacteroidetes* | | | *Chlorobi* | *Chloroflexi* | *Firmicutes* | |
|---|---|---|---|---|---|---|---|
| Class | *Bacteroidia* | *Sphingobacteriia* | *Bacteroidetes* | *Chlorobia* | *Dehalococcoidetes* | *Bacilli* | *Clostridia* |
| Order | *Bacteroidales* | *Sphingobacteriales* | *WCHB1-32* (unclassified) | *Chlorobiales* | *Order Incertae Sedis* | *Bacillales* | *Clostridiales* |
| S1 | 0.10 | 0.00 | 0.00 | 0.00 | 0.04 | 0.00 | 0.30 |
| S2 | 0.16 | 0.00 | 0.00 | 0.00 | 0.07 | 0.00 | 0.21 |
| B1 | 0.36 | 0.00 | 0.00 | 0.11 | 0.01 | 0.00 | 0.34 |
| B2 | 0.24 | 0.00 | 0.00 | 0.04 | 0.02 | 0.00 | 0.60 |
| B3 | 0.24 | 0.00 | 0.00 | 0.06 | 0.00 | 0.00 | 0.49 |
| B4 | 0.15 | 0.00 | 0.00 | 0.01 | 0.00 | 0.00 | 0.78 |
| B5 | 0.16 | 0.00 | 0.00 | 0.05 | 0.02 | 0.00 | 0.60 |
| B6 | 0.11 | 0.01 | 0.00 | 0.14 | 0.00 | 0.00 | 0.58 |
| L1 | 0.10 | 0.00 | 0.02 | 0.16 | 0.00 | 0.00 | 0.32 |
| L2 | 0.16 | 0.00 | 0.04 | 0.14 | 0.00 | 0.00 | 0.39 |
| L3 | 0.02 | 0.02 | 0.01 | 0.11 | 0.00 | 0.00 | 0.55 |
| L4 | 0.07 | 0.08 | 0.02 | 0.15 | 0.01 | 0.00 | 0.48 |
| L5 | 0.03 | 0.05 | 0.01 | 0.04 | 0.00 | 0.00 | 0.65 |
| L6 | 0.03 | 0.04 | 0.02 | 0.07 | 0.01 | 0.00 | 0.53 |
| M1 | 0.19 | 0.00 | 0.03 | 0.19 | 0.00 | 0.00 | 0.12 |
| M2 | 0.02 | 0.00 | 0.02 | 0.09 | 0.00 | 0.00 | 0.07 |
| M3 | 0.04 | 0.06 | 0.08 | 0.17 | 0.00 | 0.00 | 0.15 |
| M4 | 0.05 | 0.10 | 0.03 | 0.06 | 0.01 | 0.00 | 0.16 |
| M5 | 0.01 | 0.03 | 0.00 | 0.02 | 0.00 | 0.00 | 0.70 |
| M6 | 0.01 | 0.08 | 0.01 | 0.03 | 0.00 | 0.00 | 0.67 |
| H1 | 0.02 | 0.00 | 0.01 | 0.09 | 0.00 | 0.00 | 0.17 |
| H2 | 0.02 | 0.00 | 0.02 | 0.04 | 0.00 | 0.00 | 0.29 |
| H3 | 0.01 | 0.00 | 0.00 | 0.00 | 0.00 | 0.00 | 0.12 |
| H4 | 0.01 | 0.00 | 0.00 | 0.01 | 0.00 | 0.00 | 0.04 |
| H5 | 0.00 | 0.02 | 0.00 | 0.00 | 0.02 | 0.02 | 0.11 |
| H6 | 0.06 | 0.00 | 0.00 | 0.02 | 0.00 | 0.08 | 0.32 |
| H7 | 0.00 | 0.00 | 0.00 | 0.00 | 0.00 | 0.35 | 0.64 |
| H8 | 0.00 | 0.00 | 0.00 | 0.00 | 0.00 | 0.04 | 0.26 |
| H9 | 0.01 | 0.00 | 0.00 | 0.00 | 0.00 | 0.00 | 0.96 |

| Class | *Proteobacteria* | | | | | | | | *Spirochaetes* |
|---|---|---|---|---|---|---|---|---|---|
| | *Beta-* | | *Delta-* | | | | *Epsilon-* | *Gamma-* | *Spirochaetes* |
| Order | *Burkholderiales* | *Rhodocyclales* | *Desulfovibrionales* | *Desulfuromonadales* | *Sva0485* | *Syntrophobacterales* | *Campylobacterales* | *Oceanospirillales* | *C-2* (unclassified) |
| S1 | 0.00 | 0.01 | 0.01 | 0.12 | 0.00 | 0.03 | 0.20 | 0.00 | 0.13 |
| S2 | 0.01 | 0.02 | 0.02 | 0.14 | 0.00 | 0.04 | 0.22 | 0.00 | 0.08 |
| B1 | 0.00 | 0.01 | 0.01 | 0.03 | 0.00 | 0.06 | 0.00 | 0.00 | 0.03 |
| B2 | 0.00 | 0.01 | 0.01 | 0.03 | 0.00 | 0.02 | 0.00 | 0.00 | 0.00 |
| B3 | 0.00 | 0.00 | 0.01 | 0.01 | 0.00 | 0.03 | 0.00 | 0.00 | 0.12 |
| B4 | 0.00 | 0.00 | 0.01 | 0.00 | 0.00 | 0.01 | 0.00 | 0.00 | 0.01 |
| B5 | 0.00 | 0.00 | 0.01 | 0.00 | 0.00 | 0.01 | 0.00 | 0.00 | 0.13 |
| B6 | 0.00 | 0.00 | 0.02 | 0.01 | 0.00 | 0.01 | 0.00 | 0.00 | 0.10 |
| L1 | 0.00 | 0.00 | 0.03 | 0.10 | 0.00 | 0.09 | 0.02 | 0.00 | 0.10 |
| L2 | 0.00 | 0.00 | 0.01 | 0.07 | 0.00 | 0.05 | 0.01 | 0.00 | 0.04 |
| L3 | 0.00 | 0.00 | 0.04 | 0.06 | 0.00 | 0.03 | 0.01 | 0.00 | 0.10 |
| L4 | 0.00 | 0.00 | 0.02 | 0.03 | 0.00 | 0.04 | 0.00 | 0.00 | 0.06 |
| L5 | 0.00 | 0.00 | 0.07 | 0.03 | 0.00 | 0.02 | 0.00 | 0.00 | 0.07 |
| L6 | 0.00 | 0.00 | 0.09 | 0.06 | 0.00 | 0.02 | 0.01 | 0.00 | 0.10 |
| M1 | 0.00 | 0.01 | 0.05 | 0.13 | 0.00 | 0.11 | 0.01 | 0.00 | 0.08 |
| M2 | 0.00 | 0.00 | 0.02 | 0.37 | 0.00 | 0.05 | 0.29 | 0.00 | 0.03 |
| M3 | 0.00 | 0.00 | 0.02 | 0.20 | 0.00 | 0.03 | 0.04 | 0.00 | 0.15 |
| M4 | 0.00 | 0.00 | 0.11 | 0.18 | 0.00 | 0.05 | 0.02 | 0.00 | 0.20 |
| M5 | 0.00 | 0.00 | 0.08 | 0.06 | 0.00 | 0.03 | 0.01 | 0.00 | 0.02 |
| M6 | 0.00 | 0.00 | 0.03 | 0.07 | 0.00 | 0.02 | 0.00 | 0.00 | 0.06 |
| H1 | 0.00 | 0.08 | 0.00 | 0.11 | 0.00 | 0.02 | 0.43 | 0.00 | 0.03 |
| H2 | 0.00 | 0.06 | 0.00 | 0.05 | 0.00 | 0.01 | 0.46 | 0.00 | 0.01 |
| H3 | 0.25 | 0.18 | 0.01 | 0.09 | 0.00 | 0.00 | 0.30 | 0.00 | 0.00 |
| H4 | 0.02 | 0.01 | 0.01 | 0.28 | 0.00 | 0.00 | 0.58 | 0.00 | 0.01 |
| H5 | 0.23 | 0.01 | 0.00 | 0.03 | 0.10 | 0.01 | 0.02 | 0.01 | 0.00 |
| H6 | 0.11 | 0.00 | 0.00 | 0.03 | 0.01 | 0.01 | 0.08 | 0.14 | 0.00 |
| H7 | 0.00 | 0.00 | 0.00 | 0.00 | 0.00 | 0.00 | 0.00 | 0.00 | 0.00 |
| H8 | 0.03 | 0.61 | 0.00 | 0.00 | 0.00 | 0.00 | 0.00 | 0.00 | 0.00 |
| H9 | 0.01 | 0.00 | 0.00 | 0.00 | 0.00 | 0.00 | 0.00 | 0.00 | 0.00 |
